# Effect of Achilles tendon on kinematic coupling relationship between tarsal bones: a pilot finite element study

**DOI:** 10.1186/s13018-020-01728-0

**Published:** 2020-06-08

**Authors:** Song-Jian Li, Lei Tang, Li Zhao, Cheng-Long Liu, Yu-Bin Liu

**Affiliations:** 1 Department of Orthopaedics, Zhujiang Hospital of Southern Medical University, Guangzhou, 510280 China; 2 Department of Anatomy, Southern Medical University, Guangzhou, 510280 China; 3 Ying-Hua Medical Group of Bone and Joint Healthcare in Children, Shanghai, 200092 China

**Keywords:** Achilles tendon, Kinematic coupling, Tarsal bones, Finite element study

## Abstract

**Background:**

The procedure of percutaneous Achilles tenotomy (PAT) is an important component of the Ponseti method. However, few studies reported the influence of Achilles tendon on kinematic coupling relationship between tarsal bones. The purpose of present study was to demonstrate the effect of Achilles tendon on the kinematic coupling relationship between tarsal bones, and to illustrate how kinematic coupling relationship between tarsal bones works in term of finite element analysis.

**Methods:**

A three-dimensional finite element model of foot and ankle was constructed based on the Chinese digital human girl No.1 (CDH-G1) image database using the software of mimics, Geomagic studio, HyperMesh, and Abaqus. The last manipulation of the Ponseti method before the procedure of PAT was simulated. The talus head and the proximal tibia and fibula bone were fixed in all six degrees of freedom, and the outward pressure was added on the first metatarsal head to investigate the kinematic coupling relationship between tarsal bones.

**Results:**

The least relationship of kinematic coupling between tarsal bones was found in calcaneus. Stress concentration was mainly observed at the navicular, talus and the medial malleolus. The difference in displacement of the navicular was only found with the Achilles tendon stiffness of 0 N/mm and others. No difference in the navicular displacement was found in the stiffness of Achilles tendon between 40, 80, 200, 400, and 1000 N/mm. The maximum displacement of navicular was observed at the ankle position of PF-20° (plantar flexion-20°). The difference in displacement of the navicular was greater at the ankle position of PF-20° with the Achilles tendon stiffness of 0 N/mm than that at the ankle position of PF-40° with the Achilles tendon stiffness of 40 N/mm.

**Conclusions:**

Based on the findings from this study, it was demonstrated that the Achilles tendon existence or not and ankle position had great influence, while increased stiffness of Achilles tendon had no influence on kinematic coupling relationship between tarsal bones. For the cases with severe equinus, earlier implementation of PAT procedure (with the purpose of release the Achilles tendon and reduce the degree of ankle plantar flexion) may be beneficial to the deformity correction.

## Background

Specific maneuver is one of the core components of the Ponseti method, which has been widely applied for the management of congenital clubfoot [1–4]. The kinematic coupling relationship between tarsal bones was the basis for simultaneous correction of multiple deformity components including cavus, equinus, varus, and adductus [3–6]. During the Ponseti manipulation, the thumb is placed on the lateral aspect of the talus head as a fulcrum while outward pressure is exerted on the first metatarsal and first cuneiform [3, 7]. When the forefoot is abducted laterally, the anterior portion of the calcaneus will be displaced outward underneath the head of the talus, and thus the varus will be corrected simultaneously. The realignment of the calcaneocuboid, the talocalcaneonavicular, and the posterior talocalcaneal joints is produced by means of the kinematic coupling relationship between tarsal bones [5, 6].

In anatomy, Achilles tendon connected the soleus and gastrocnemius muscles to the calcaneus to allow plantar flexion of the foot at the ankle. Percutaneous Achilles tenotomy (PAT) is an important component of the Ponseti method. It is indicated when 15° of ankle dorsiflexion is not obtained after other deformities fully corrected [3, 4, 8]. The reported success rate following PAT ranged from 73 to 100% [8–10], and there are still left many patients who probably do not respond adequately to the procedure of PAT. In our reported study, the initial correction rate was only 87% using PAT procedure [1]. Few papers reported the management of these “failed” PAT patients, as the correction was different from the primary cases [11]. Surgical options, such as posteromedial releases [12], gastrocsoleus fascial release, Achilles Tendon lengthening [13], and various osteotomy, were reported according to age and severity of deformity. Mehtani et al. [14] have described a “modified Ponseti method” for achieving better ankle dorsiflexion in neglected children. Agarwal et al. [15] reported the “extended Ponseti method” of continued stretching casts with a weekly change for a further 3 weeks for failed tenotomy in idiopathic clubfeet. All the reported “modified Ponseti protocol” showed good results in salvaging failed tenotomy cases. However, no studies reported the underlying cause for the “failed” PAT patients and explore the deep effect of Achilles tendon on Ponseti maneuver. As the core principle of the Ponseti method, no studies reported the influence of Achilles tendon on kinematic coupling relationship between tarsal bones. In present study, three-dimensional finite element model was established to explore the effect of Achilles tendon on the kinematic coupling relationship between tarsal bones while outward pressure was exerted on the first metatarsal. We aimed to explore how kinematic coupling relationship between tarsal bones works and illustrate the stress and displacement distribution for tarsal bones when outward pressure was added. Also, the clubfoot after initial correction can be considered the normal foot [1, 2, 5]. We built a normal model of foot and ankle to clarify the relative motion and displacement between tarsal bones when outward pressure was added on the first metatarsal. We hypothesized that the Achilles tendon plays an important role in maintaining the effectiveness of kinematic coupling relationship between tarsal bones.

## Methods

### Model establishment

The geometry of the foot model was reconstructed using the left foot obtained from the Chinese digital human girl No.1 (CDH-G1) image database (specimen of the full-term female infant with body weight of 3.2 kg and the body length of 39.2 cm, and the slice thickness of 0.1 mm). The contours of the distal tibia and fibular, the whole talus and calcaneus, the cuboids, the navicular, and the five metatarsal were extracted using the software of Photoshop CS3 (Adobe Company, San Jose, CA, USA) and the geometry of these structures was rebuilt with the software of Mimics 17 (Materialise software, Leuven, Belgium). A different threshold limit was chosen to distinguish the cartilage and bone components in the gray-scale images; the operation of segmentation was performed. The 3D geometrical objects were calculated after the segmentation operation, and then imported into the software of Geomagic studio 12 to repair and smooth the irregularity of the model. The geometric model was then converted into the software of Hypermesh 13.0 (Altair Company, Troy, MI, USA) and meshed with tetrahedron elements, and then a solid mesh model was obtained. Finally, the solid mesh model generated from HyperMesh 13.0 was then imported into the software of Abaqus 6.12 (Dassault Systemes Simulia Company, Providence, RI, USA) for finite element analysis.

### Material properties

According to the Ponseti method, clubfoot treatment should be started soon (7 to 10 days) after birth [6, 8]. The material properties of the model parts were referred to those from the data available in previous literature with a reference to the age. Bones and cartilages in infant foot were idealized as being homogeneous, isotropic, and linearly elastic. The Young’s modulus of the bone and cartilage for newborn baby were assigned as 38 MPa and 2.3 MPa, respectively, while the Poisson’s ratio was 0.3 for bone and 0.4 for cartilage [16]. In present study, the distal tibia and fibular, and five metatarsal bones were assigned as bone material properties, and the whole talus, calcaneus, three cuboids and navicular bone were assigned as cartilage material properties for the consideration of the infant foot anatomy and radiographic characteristics. The ligaments were modeled as linear springs. As reported in the previous studies, the use of linear links to simulate ligaments was found to be adequate [17, 18]. As no study was found to report the stiffness of ligament in the infant foot, we defined the stiffness of infant foot ligaments based on the adult stiffness in foot ligament and multiple relationships calculated from the research of mechanical properties of cervical spine ligaments between a 14-year-old child and newborn baby [19, 20]. Luck et al. [19] reported that the stiffness of the whole cervical spine (WCS) was 6.8 N/mm for the 11-day-old baby and 70.1 N/mm for the 14-year-old child. The Achilles tendon stiffness was reported as 306 to 530 N/mm in adults [21, 22]. No previous study reported the Achilles tendon stiffness in newborn; we calculated the Achilles tendon stiffness of 40 N/mm as normal Achilles tendon stiffness for newborn baby based on data reported previously [19]. The insertion and original sites and the number of ligaments were determined based on anatomical locations and previous literature [20]. A total of 28 ligaments and the Achilles tendon were modeled as linear springs with assigned stiffness values, as shown in Table 1. The contact behavior between the articulating surfaces was considered frictionless [23] and surface-to-surface contact behavior with the sliding formulation of small sliding [24]. The simulated axis of ankle motion was nearly consistent with the line of medial and lateral malleolus cusp, outward-inclined about 8° in coronal plane and external rotation about 6° in transection plane [25]. The micro motion between tarsal bones was ignored in the present study. A mesh convergence study was performed using five different mesh sizes, from 1 to 5 mm. The optimum mesh size for the bone and cartilage was set as 3 mm. The numerical model used in this study contains about 339,310 elements and 69,144 nodes.

**Table 1:** Stiffness of ligaments

Ligaments represented in the models	Connected bones	Stiffness (N/mm)^a^
Interosseous membrane (4 ligaments)	Tibia–fibula	40
Anterior tibiofibular (distal)	Tibia–fibula	7.8
Posterior tibiofibular (distal)	Tibia–fibula	10.1
Anterior tibiotalar	Tibia–talus	7
Posterior tibiotalar	Tibia–talus	8
Tibiocalcaneal	Tibia–calcaneus	12.2
Tibionavicular	Tibia–navicular	4
Interosseous talocalcaneal	Talus–calcaneus	7
Lateral talocalcaneal	Talus–calcaneus	7
Medial talocalcaneal	Talus–calcaneus	7
Posterior talocalcaneal	Talus–calcaneus	7
Anterior talofibular	Talus–fibula	14.2
Posterior talofibular(2 ligaments)	Talus–fibula	8
Calcaneofibular ligament	Calcaneus–fibula	12.7
Dorsal talonavicular (2 ligaments)	Talus–navicular	7
Calcaneonavicular (dorsal and plantar)	Calcaneus–navicular	7
Calcaneocuboid (dorsal and short plantar)	Calcaneus–cuboid	7
Cuboideonavicular (dorsal and plantar)	Cuboid–navicular	7
Cuneonavicular (dorsal and plantar)	Cuneiforms–navicular	7
Intercuneiform (dorsal and plantar)	Lateral-intermediate–medial cuneiform	7
Tarsometatarsal (dorsal and plantar)	Cuneiforms–metatarsals	7
Metatarsal (dorsal and plantar)	1st–2nd–3rd–4th–5th metatarsal	7
Long plantar	Calcaneus–metatarsals	7
Achilles tendon	-	40

^a^Calculation based on the foot ligaments stiffness value and multiple relationships according to the cervical ligament parameters reported in 14-year-old child and newborn baby [19, 20]

### Maneuver simulation

For better illustration of kinematic coupling relationship between tarsal bones, the distal tibia and fibula bone and the head of talus were fixed in all six degrees of freedom during the whole test. Equinus was defined as the increased stiffness of Achilles tendon and increased plantar flexion of the ankle in present finite element model. Earlier PAT was defined as early PAT procedure before forefoot adequate abduction (abducted to 60° to 70° without pronation) with ankle dorsiflexion remains less than 10°. The ankle plantar flexion was set at 20° to simulate the equinus deformity for exploring the role of Achilles tendon. The outward pressure was added on the first metatarsal head to observe the stress distribution and displacement of navicular bone. We defined the magnitude of kinematic coupling of tarsal bones as the displacement shift of each tarsal bone after outward pressure added in present finite element model.

## Results

### Validation

**Fig. 1:**
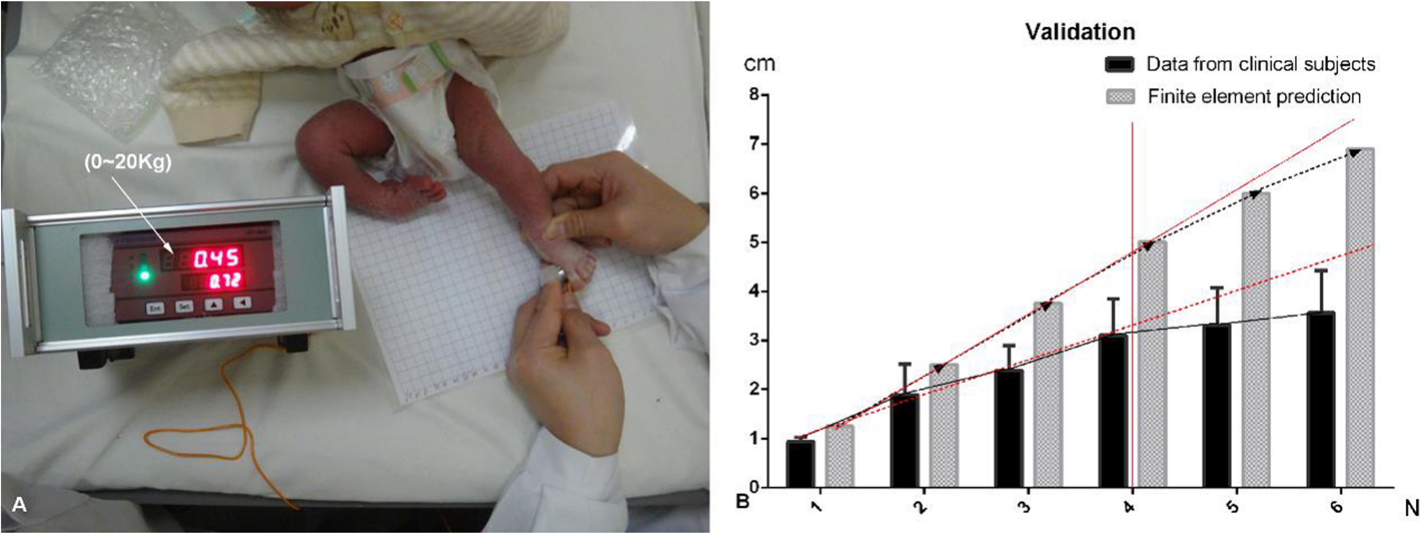
Outward pressure was measured using mini pressure sensor (**a**). The validation of the established model was presented in **b** (the red line indicates the reference line)

**Fig. 2:**
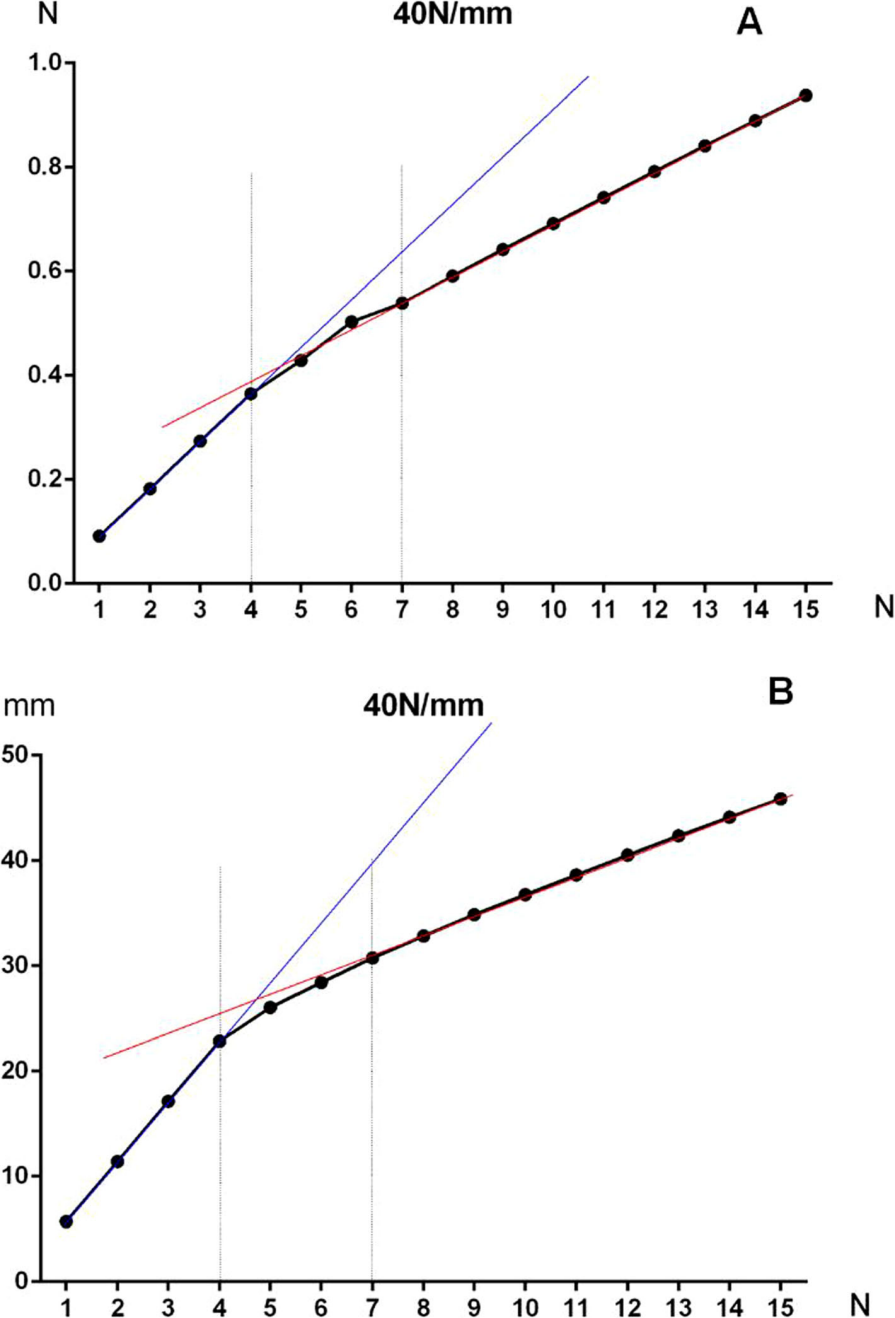
The stress and displacement of navicular bone changed with the pressure load added on the first metatarsal from 1 to 15 N. **a** The stress distribution of navicular bone. **b** The displacement distribution of navicular bone

The validation of the established model was to investigate the relationship between the displacement and pressure added on the first metatarsal head (Fig. 1). The displacement of the first metatarsal head was compared between finite element prediction and clinical data collected using mini pressure sensor (measuring range from 0 to about 20 kg, Fig. 1a). The data from the clinical test was collected from the 6 feet in unilateral clubfeet. A linear positive relationship was presented in both groups with the outward pressure added from 1 to 4 N, and deviation was found when the pressure was added from 4 to 6 N (Fig. 1b). The stress and displacement relationship of the navicular bone was presented in the Fig. 2. The results of self-validation of finite element model showed that the stress and displacement increased linearly as the pressure added gradually on the first metatarsal head (from 0 to 4 N), then the stress and displacement increment of navicular bone (the slope of the line) was gradually reduced as the gradient pressure added on the first metatarsal head from 4 to 7 N (Fig. 2). The linear increase of stress and displacement was observed with constant slope of line when outward pressure added more than 7 N (Fig. 2). Also, it was previously reported that the applied force on the first metatarsal was 4.2 N during the manipulation based on an instrumented clubfoot model [26]. There was the similar stress distribution and intensity on the first metatarsal head (maximum from 4 to 7 N) presented in this study.

### Stress and displacement distribution for tarsal bones

**Fig. 3:**
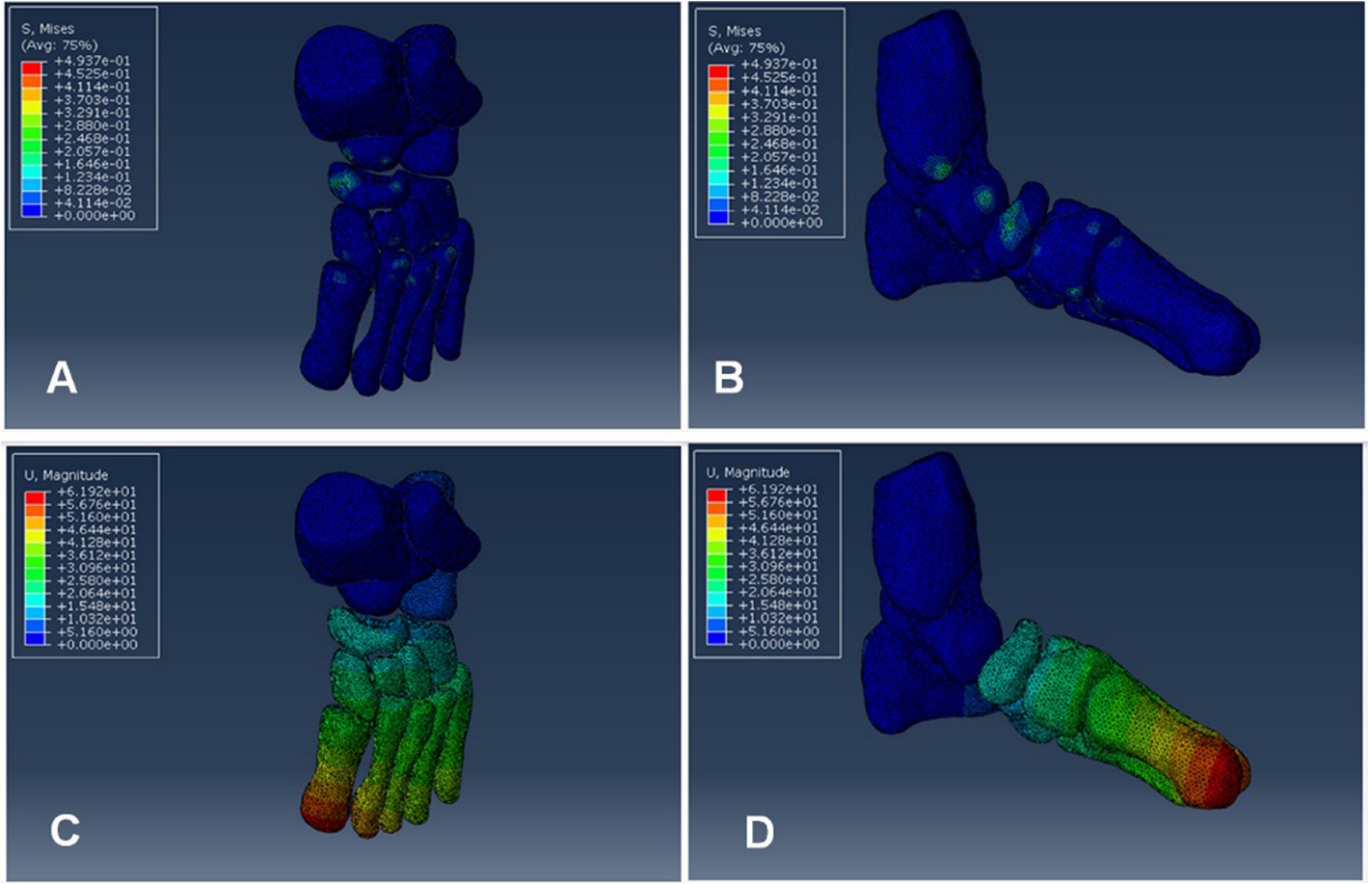
von Mises stress and displacement distribution was revealed for the whole finite element model. **a**, **b** The stress distribution of whole foot model. **c**, **d** The displacement distribution of whole foot model

von Mises stress and displacement distribution of the whole model with the Achilles tendon stiffness of 40 N/mm was presented in Fig. 3 under outward pressure of 3 N. The maximum von Mises stress was observed at the navicular, talus and medial malleolus, while the navicular bone was the main stress concentration region (Fig. 3a, b). The maximum displacement was observed at the first metatarsal bone, and the displacement decreased gradient from distal to proximal of whole model (Fig. 3c, d). The minimum displacement of tarsal bones was observed at calcaneus bone. It is indicated that the calcaneus bone had the least kinematic coupling relationship between tarsal bones.

### Effect of Achilles tendon

**Fig. 4:**
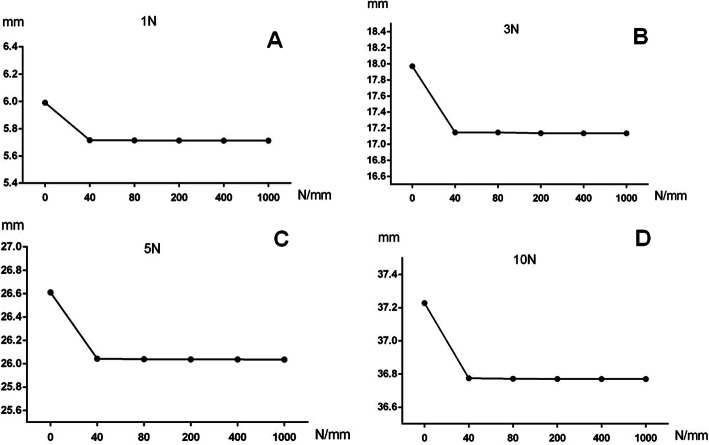
Greater difference was found in the navicular displacement with Achilles tendon between 0 N/mm and anyone of others. Increased stiffness of Achilles tendon seemed to have no influence on the displacement of navicular bone

The relationship between the stiffness of Achilles tendon and the kinematic coupling relationship between tarsal bones was calculated and presented in Fig. 4 at the ankle position of plantar flexion (PF)-20°. There was a greater difference in the displacement of navicular bone when the stiffness of Achilles tendon ranged between 0 N/mm and any one of others, such as 40, 80, 200, 400, and 1000 N/mm. No difference in the navicular displacement was found in the stiffness of Achilles tendon between 40, 80, 200, 400, and 1000 N/mm including the outward pressure of 1 N, 3 N, 5 N, and 10 N. It was supposed that the increased stiffness of Achilles tendon had no influence, while the presence or absence of Achilles tendon had great influence on the kinematic coupling relationship of tarsal bones.

### The common effect of ankle position and Achilles tendon

**Fig. 5:**
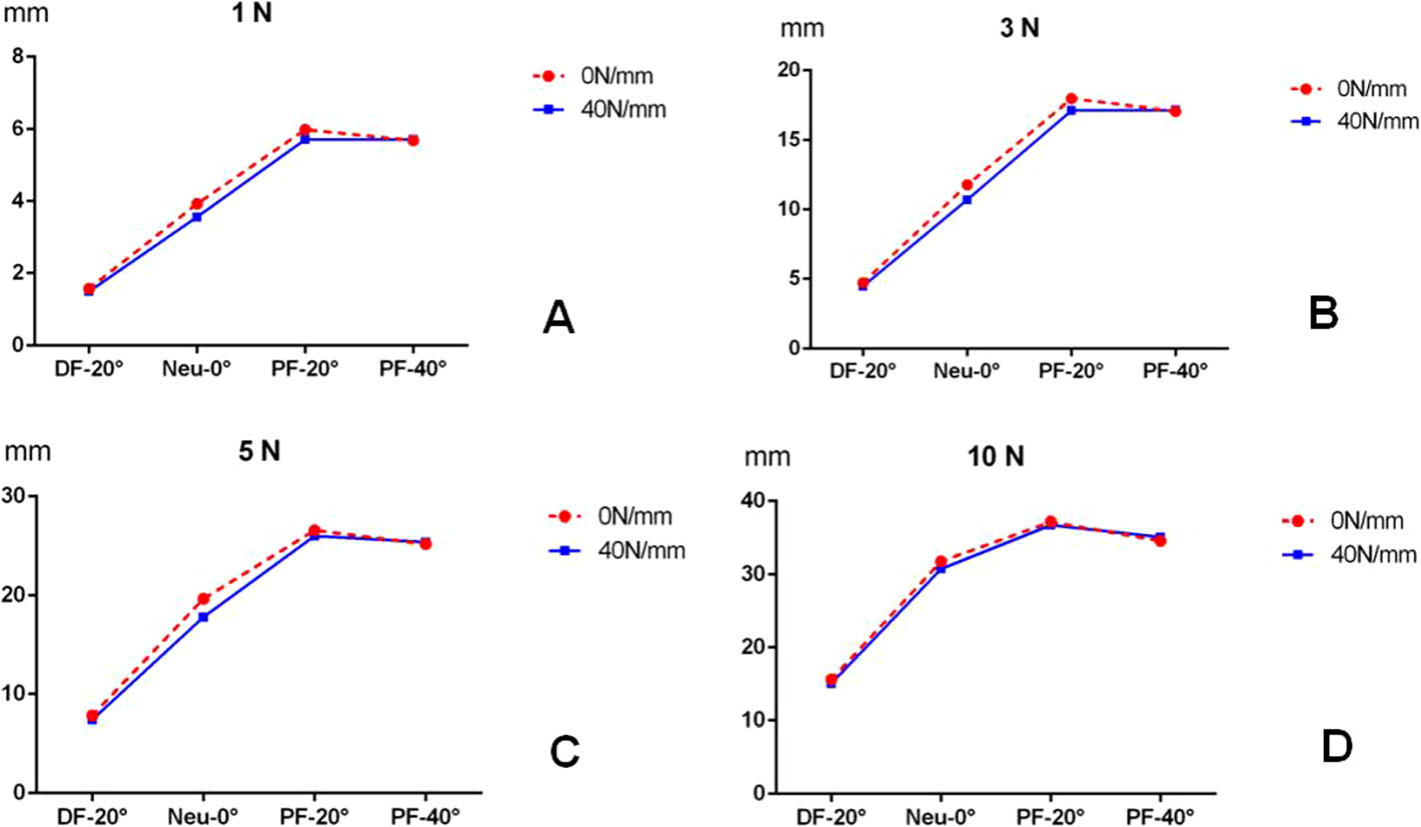
The common effect of ankle position and Achilles tendon was presented. The maximum displacement of navicular bone was observed at the ankle position of PF-20°

**Fig. 6:**
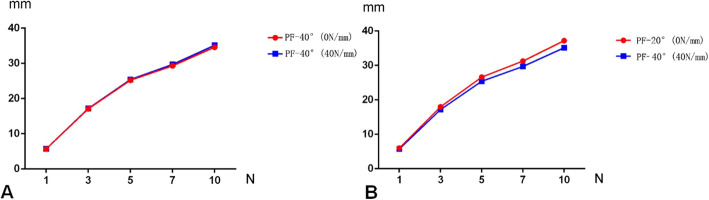
The displacement was greater at the ankle position of PF-40° with the Achilles tendon stiffness of 40 N/mm than 0 N/mm (**a**). Decreased displacement was found in the ankle position of PF-40° with the Achilles tendon stiffness of 40 N/mm than the ankle position of PF-20° with the Achilles tendon stiffness of 0 N/mm (**b**)

The correlation between the ankle position and the kinematic coupling relationship between tarsal bones was calculated and presented in Fig. 5. The maximum displacement of navicular bone was identified at the ankle position of PF-20°. In case of the ankle position of PF-40° and dorsiflexion-20° (DF-20°), the displacement of navicular was reduced in comparison with the ankle position of PF-20°. Greater displacement of navicular was observed at the Achilles tendon stiffness of 0 N/mm than that of 40 N/mm at the ankle position of DF-20°, neutral-0° (Neu-0°) and PF-20°, while reduced displacement of navicular bone was observed at the ankle position of PF-40° with the Achilles tendon stiffness of 0 N/mm than 40 N/mm (Fig. 5). The displacement of navicular was greater at the ankle position of PF-40° with the Achilles tendon stiffness of 40 N/mm than 0 N/mm (Fig. 6a). However, the decreased displacement of navicular bone was found in the ankle position of PF-40° with the Achilles tendon stiffness of 40 N/mm than the ankle position of PF-20° with the Achilles tendon stiffness of 0 N/mm (Fig. 6b). It was supposed that earlier implementation of PAT procedure to reduce the degree of ankle plantar flexion may enhance kinematic coupling relationship of tarsal bones in case of severe equinus deformity.

## Discussions

Equinus deformity was an important component of clubfoot deformity and contracture of Achilles tendon contributed greatly to this deformity. The kinematic coupling relationship between tarsal bones was core principle for simultaneous correction of multiple deformity components [5, 6]. In some cases of clubfoot with severe equinus deformity, some authors recommended the posterior capsulotomy to obtain adequate correction for these challenging cases at the sagittal plane [27, 28]. Therefore, we have to reevaluate the role of Achilles tendon in the Ponseti method for the clubfoot management. Unfortunately, little was known about the correlation between Achilles tendon and kinematic coupling relationship between tarsal bones. For further exploring the intrinsic mechanism for the role of the Achilles tendon in the kinematic coupling relationship between tarsal bones, the CDH-G1 image database was applied to establish the 3D foot model and to understand the effect of the of Achilles tendon in response to the Ponseti maneuver.

The finite element model was validated mainly using the data from cadaveric experiment with similar settings, published studies data or self-validation of established model for the comparison [29–31]. To our knowledge, no previous studies have reported the biomechanics of simulated Ponseti maneuver in terms of either cadaveric experiment or finite element analysis. The established model of present study was validated and compared to the clinical data collected from 6 unilateral clubfoot cases tested using mini pressure sensor. The results showed that practical manipulation was well simulated in the finite element model under the pressure added less than 4 N, while variation of displacement was found under the pressure load added from 4 to 6 N. This may be attributed to the simplification of the established model. The ligaments in the present study were simulated as linear springs, and the influence of capsule and muscle in the foot was disregarded in present study. These differences lead to the absence of “transitional phase” and replacement of reduced increment of displacement (slope of the line) in finite element model. The stress and displacement of navicular bone revealed that the pressure added on the first metatarsal head from 4 to 7 N was an important “transitional phase” for the clinical effectiveness in the process of Ponseti maneuver. It was supposed that the maximum stress may range from 4 to 7 N to avoid the injury of soft tissues. Also, it was previously reported that the applied force on the first metatarsal was 4.2 N during the manipulation based on an instrumented clubfoot model [26]. There was the similar stress distribution and intensity on the first metatarsal head (maximum from 4 to 7 N) presented in this study.

The maximum stress distribution was found to be mainly concentrated on the talus and navicular. This supposedly means that talonavicular joint was the most important articulation for deformity correction during the process of Ponseti maneuver. The pathoanatomy of clubfoot showed that the navicular bone was wedge-shaped and medially displaced, while the medial tuberosity of the navicular was approached to the medial malleolus [6, 7]. As reported by Pirani et al [32], the correction by means of the Ponseti method included not only the relocation of the abnormal relationships between the tarsal bones, but also the remodeling of the individual tarsal osteochondral anlages. In present study, the stress concentration was found on the navicular bone when outward pressure was added. The maximum stress distribution was mainly focused on the insertion of tibionavicular ligaments. This presumably indicates that the manipulation in the Ponseti method has mainly contributed to stretching the tibionavicular ligament. This was consistent with the findings in the pathoanatomy of clubfoot that the tibionavicular ligament was very thick and short [3, 5, 6]. The kinematic coupling relationship was observed in that calcaneus, cuboid, three cuneiform, and five metatarsal bones simultaneously moved laterally as the outward pressure added on the first metatarsal head. This presumably explains that Ponseti maneuver was an appropriate and effective way to correct all the deformity components. The minimum displacement was observed at calcaneus bone, which indicated that the calcaneus bone had the least movement in response to the kinematic coupling relationship of tarsal bones. Herein, it was recommended that the forefoot should be abducted adequately to correct the hind-foot varus in clinical practice.

The effect of Achilles tendon revealed that the navicular displacement at the Achilles tendon stiffness of 0 N/mm was greater than that of 40, 80, 200, 400, and 1000 N/mm. Also, the decreased displacement of navicular bone was found in the ankle position of PF-40° with the Achilles tendon stiffness of 40 N/mm than the ankle position of PF-20° with the Achilles tendon stiffness of 0 N/mm. In clinical practice, it was easy to understand that the PAT procedure had the effect not only on the existence of Achilles tendon but also on the ankle position. Based on the results, it was supposed that earlier implementation of PAT procedure may enhance the kinematic coupling relationship of tarsal bones and facilitate the deformity correction. The common effects of Achilles tendon and ankle position revealed that more displacement of navicular bone was observed at the ankle position of PF-20° compared with that at the ankle position of Neu-0° and PF-40° (Fig. 5). It is supposed that the most effective position of kinematic coupling relationship of tarsal bones may be related to the ankle position of PF-20°. The explanation may be attribute to the “locks and unlocks mechanism” between transverse tarsal joint. When the ankle position was set to Neu-0° and DF-20°, more stability of the ankle was triggered for the locking of transverse tarsal joint. The kinematic coupling relationship of tarsal bones was disturbed because of the stability of the ankle. The reduced displacement was also found in the ankle position PF-40°, which may be caused to the excessive unlock of transverse tarsal joint, and then reduced the kinematic coupling relationship of tarsal bones. The stiffness of Achilles tendon stiffness in 0 N/mm had more navicular displacement than that of 40 N/mm at the positions of DF-20°, Neu-0° and PF-20°, while no benefit was obtained for the ankle position of PF-40°. It was supposed that the effectiveness of kinematic coupling relationship between tarsal bones may benefit from earlier implementation of PAT procedure at the positions of DF-20°, Neu-0°, and PF-20°, except for the ankle position of PF-40°. However, the equinus deformity was corrected from quite severe to less severe position using the protocol of earlier PAT procedure (for example, less than PF-20°), the manipulation effectiveness may be enhanced (Fig. 6). This was consistent with clinical experience that it was difficult to correct the severe equinus deformity (PF-40°) in some challenging case before the procedure of PAT.

The merit of present study was that the effects of Achilles tendon and the kinematic coupling relationship of tarsal bones were firstly demonstrated in terms of finite element method. The geometry of the foot and ankle model was reconstructed from CDH-G1 image database, which had better recognized contour of foot cartilage and bone than that of magnetic resonance imaging (MRI), and provided more and precise information of foot tarsal bone in newborn. The incidence of clubfeet is higher in male. The established model may be better from the Chinese digital human boy. However, the aim of preset study was to focus on the kinematics of relationship between Achilles tendon and tarsal bones, not on the biology of the deformity. The contours of tarsal bone were extracted and ligaments were modeled as linear springs for the established model in present study. So, we think there was little or no deviation to the results. Another limitation was that we applied the normal foot model in a newborn to simulate the Ponseti maneuver; the variance did exist between clubfoot and normal foot model. However, we know that the clubfoot could be considered the normal foot after the Ponseti method correction [3–6]. The context of model simplifications and settings in present study was another limitation. We only extracted the information of cartilage and bone to build the 3-D foot model, and the Achilles tendon and ligaments were modeled with springs that may result in stress concentration at the insertions.

In conclusion, it was demonstrated that Achilles tendon has great effect on kinematic coupling relationship between tarsal bones, and the least kinematic coupling relationship between tarsal bones was found in the calcaneus. The increased stiffness of Achilles tendon had no influence on kinematic coupling relationship between tarsal bones. The ankle position of PF-20° was the effective position where the kinematic coupling relationship of tarsal bones was revealed. For the cases with severe equinus, earlier implementation of PAT procedure, with the purpose of releasing the Achilles tendon and reducing the degree of ankle plantar flexion, may be beneficial to the deformity correction.

## Data Availability

The datasets used and/or analyzed during the current study are available from the corresponding author on reasonable request.

## Abbreviations

PAT: Percutaneous Achilles tenotomy
CDH-G1: Chinese digital human girl No.1
PF: Plantar flexion
DF: Dorsiflexion-20°
Neu: Neutral
MRI: Magnetic resonance imaging
